# The Voice of the Body: Why AI Should Listen to It and an Archive

**DOI:** 10.34133/cbsystems.0005

**Published:** 2023-01-16

**Authors:** Kun Qian, Bin Hu, Yoshiharu Yamamoto, Björn W. Schuller

**Affiliations:** ^1^ School of Medical Technology, Beijing Institute of Technology, Beijing 100081, China.; ^2^ Educational Physiology Laboratory, Graduate School of Education, The University of Tokyo, Tokyo 113-0033, Japan.; ^3^ GLAM—Group on Language, Audio, & Music, Imperial College London, London SW7 2AZ, UK.; ^4^ Chair of Embedded Intelligence for Health Care and Wellbeing, University of Augsburg, 86159 Augsburg, Germany.

## Abstract

The sound generated by body carries important information about our health status physically and psychologically. In the past decades, we have witnessed a plethora of successes achieved in the field of body sound analysis. Nevertheless, the fundamentals of this young field are still not well established. In particular, publicly accessible databases are rarely developed, which dramatically restrains a sustainable research. To this end, we are launching and continuously calling for participation from the global scientific community to contribute to the Voice of the Body (VoB) archive. We aim to build an open access platform to collect the well-established body sound databases in a well standardized way. Moreover, we hope to organize a series of challenges to promote the development of audio-driven methods for healthcare via the proposed VoB. We believe that VoB can help break the walls between different subjects toward an era of Medicine 4.0 enriched by audio intelligence.

## Introduction

**Fig. 1.**
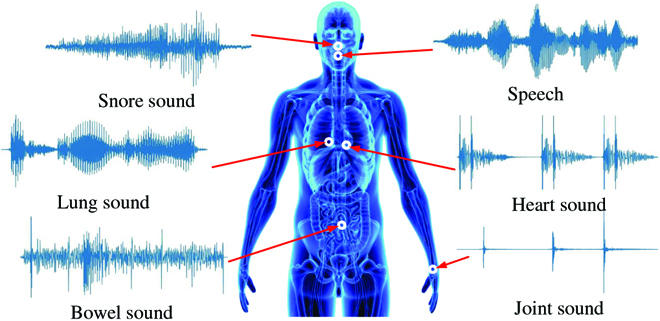
An overview of the body sound analysis. Speech data are studied widely for measuring the human health status, whereas the body sound data are not thoroughly studied for healthcare applications. The different characteristics of various body sounds can be summarized as follows: First, the vibration mechanism is unique between each kind of body sound. Second, the acoustic features (e.g., the main energy distribution of the spectrum) are varied. These body sounds can be collected via the prevalent microphone equipment and/or wearable devices. The sounds generated by our human body can reveal the health status in both physical and mental terms.

Auscultation, an important medical skill, has been serving as a cheap, convenient, and efficient method for more than one century. The early work can be traced back to the 1900s, when Cannon [1] studied the sound generated by the stomach and intestines. Besides, auscultation is always playing a crucial role in the diagnosis methods of the traditional Chinese medicine [2]. We can understand that the body sound carries the information reflecting the status of our health status for both physiology and psychology (see Fig. 1). Therefore, analysis of the characters inherited in the body sound can help disclose the conditions of the organs and/or the mind. In this era of artificial intelligence (AI), we can realize auscultation in a computational way by leveraging the power of computer audition (CA), which has already made and is continuously achieving a plethora of promising progresses in the healthcare domain [3].

On the one hand, tremendous efforts from the community of CA have been given to the field of digital health. In the past decade, it is increasingly developing the state-of-the-art technologies to enable a fast, cheap, and efficient diagnosis of diseases via body sound analysis. We can see the successes achieved, e.g., in the detection of sleep disorders [4], cardiovascular diseases (CVDs) [5], lung diseases [6], bowel diseases [7], musculoskeletal injury [8], and even the ongoing coronavirus disease 2019 (COVID-19) pandemic [9]. On the other hand, the power and potential of CA in medicine are still underestimated if we compare it to its main counterpart, computer vision (CV). The main challenges on the way to break the walls between CA and medical technology are as follows: First, open accessible databases are severely lacking. Most of the existing studies were executed on privately used databases, which dramatically restrains a reproducible and sustainable research in this field. The main reason for data scarcity is the difficult annotation process. Unlike speech data annotation, body sound annotation requires medical knowledge and professional training. In addition, how to assure according data privacy is another important factor that needs to be taken into account. The methods are thereby specifically designed to analyze body sounds rather than speech. Moreover, standards of data acquisition, annotation, and partition are not always consistent throughout the reported works. This limitation may impede a fair comparison between algorithms and models. Second, public challenges are rarely developed. We can see that the successful PhysioNet/CinC Challenge [10] has been continuously attracting attention to heart sound classification tasks, whereas its effect is limited to the CVD community. A comprehensive archive is the prerequisite to broaden the frontiers of knowledge in discovering the human body sounds in the era of AI-empowered Medicine 4.0.

## Voice of the Body

To address the above-mentioned challenges, we are continuously collecting open accessible body sound databases to build a comprehensive archive, i.e., the voice of the body (VoB) (http://www.vob-bit.org/). The VoB archive will be a public platform run for disseminating, exchanging, and inspiring brave new ideas toward the emerging technologies on body sound analysis. We will maintain this archive by the following guidance: First, any prospective contributors can apply to submit their well-established databases to VoB. We will examine the data quality, check the license, and receive the permission from the owners to publish the data information online. A well-written database introductory paper (may or may not be published) is appreciated. Second, we will provide benchmark experiments by our open source toolkits (e.g., openSMILE [11]) to set up the baselines. This benchmark work can be reproducible and comparable by any other database users. Third, the users who share interest in VoB databases can freely apply for one and/or all of the published databases by signing a license. They can train and tune their models by only using the train and development sets. After they submit the predicted results made by their optimized models in the VoB website, a response of their results on the test set will be automatically sent to their registered e-mail addresses. The main advantages of the VoB can be summarized as follows: First, to the best of our knowledge, VoB is the first publicly accessible platform that aims to collect all types of body sound data. This valuable archive can enhance the deep exploration of the body sounds by a broad community of audience who share according interests. Second, unlike the existing body sound databases, VoB will set a series of standards in data acquisition, annotation, and model training/testing. These standards shall assure a fair comparison between different algorithms/models. Third, the VoB platform shall—beyond providing a rich archive for data scientists to develop their novel solutions—also provide a strong professional training resource for medical training.

## Outlook

First, from now on, we are calling for contributions from the global scientific community who share the same interests in body sound analysis. If you may not be familiar with AI and/or CA, please feel free to contact us to receive help on how to establish a standard database. In the future, we will organize series of challenges at prestigious suited conferences (e.g., ISCA INTERSPEECH, IEEE ICASSP, and ACM Multimedia) by presenting the VoB tasks. In addition, a comprehensive summary of the lessons learnt from the challenges and/or other contributions by using the VoB databases will be published.

Then, for deep learning methods, models are always “hungry for data.” However, data scarcity is an ever-lasting difficulty in almost all the subfields of digital health. In particular, assuring any accurate annotation of the body sound needs tremendous experts’ knowledge, which renders the annotation work expensive and time-consuming. Therefore, seeking for solutions on fully exploiting the information from a large amount of unlabeled data is important.

Furthermore, explainability is a crucial factor that should never be ignored when building an AI system in the healthcare scenario. Nevertheless, the state-of-the-art works on body sound analysis are insufficient in building up explainable AI systems. This limitation not only hampers the acceptance of the promising results from an ethical perspective but also restrains the progress of the algorithms/models. We hope that the VoB archive can reach milestone contributions toward reaching an explainable AI (XAI) in exploring the secrets of the body sound.

Last but not least, it is reasonable to think that body sound can be a novel digital phenotype for measuring the physical status of our body. In fact, the existing studies have already demonstrated the potential. But how could the body sound reflect our mental health status? For instance, the “brain–gut interactions” are related to the presence of anxiety or depression [12]. A related problem that needs to be answered is if a bowel sound could be a good indicator for us to understand the status of our mind?

## References

[1] Cannon WB. Auscultation of the rhythmic sounds produced by the stomach and intestines. Am J Physiol. 1905;14(4):339–353.

[2] Lukman S, He Y, Hui S-C. Computational methods for traditional Chinese medicine: A survey. Comput Methods Prog Biomed. 2007;88(3):283–294. 10.1016/j.cmpb.2007.09.008 17983685

[3] Qian K, Li X, Li H, Li S, Li W, Ning Z, Yu S, Hou L, Tang G, Lu J, et al. Computer audition for healthcare: Opportunities and challenges. Front Digit Health. 2020;2:5. 34713018 10.3389/fdgth.2020.00005 PMC8521830

[4] Qian K, Janott C, Schmitt M, Zhang Z, Heiser C, Hemmert W, Yamamoto Y, Schuller BW. Can machine learning assist locating the excitation of snore sound? A review. IEEE J Biomed Health Inform. 2021;25(4):1233–1246. 32750978 10.1109/JBHI.2020.3012666

[5] Clifford GD, Liu C, Moody B, Millet J, Schmidt S, Li Q, Silva I, Mark RG. Recent advances in heart sound analysis. Physiol Meas. 2017;38:E10–E25. 28696334 10.1088/1361-6579/aa7ec8 PMC11460977

[6] Palaniappan R, Sundaraj K, Ahamed NU. Machine learning in lung sound analysis: A systematic review. Biocybern Biomed Eng. 2013;33(3):129–135.

[7] Allwood G, Du X, Webberley KM, Osseiran A, Marshall BJ. Advances in acoustic signal processing techniques for enhanced bowel sound analysis. IEEE Rev Biomed Eng. 2019;12:240–253. 30307875 10.1109/RBME.2018.2874037

[8] Teague CN, Hersek S, Töreyin H, Millard-Stafford ML, Jones ML, Kogler GF, Sawka MN, Inan OT. Novel methods for sensing acoustical emissions from the knee for wearable joint health assessment. IEEE Trans Biomed Eng. 2016;63(8):1581–1590. 27008656 10.1109/TBME.2016.2543226

[9] Deshpande G, Batliner A, Schuller BW. AI-based human audio processing for COVID-19: A comprehensive overview. Pattern Recogn. 2022;122(108289):1–10. 10.1016/j.patcog.2021.108289 PMC8404390 34483372

[10] Liu C, Springer D, Li Q, Moody B, Juan RA, Chorro FJ, Castells F, Roig JM, Silva I, Johnson AE, et al. An open access database for the evaluation of heart sound algorithms. Physiol Meas. 2016;37(12):2181–2213. 27869105 10.1088/0967-3334/37/12/2181 PMC7199391

[11] Eyben F, Weninger F, Gross F, Schuller B, Recent developments in openSMILE, the Munich open-source multimedia feature extractor. Paper presented at: MM '13: Proceedings of the 21st ACM International Conference on Multimedia; 2013 October; Barcelona, Spain; p. 835–838.

[12] Gracie DJ, Hamlin PJ, Ford AC. The influence of the brain–gut axis in inflammatory bowel disease and possible implications for treatment. Lancet Gastroenterol Hepatol. 2019;4(8):632–642. 31122802 10.1016/S2468-1253(19)30089-5

